# Investigation of Physical Properties of Polymer Composites Filled with Sheep Wool

**DOI:** 10.3390/polym16050690

**Published:** 2024-03-02

**Authors:** Martin Vasina, Premysl Straznicky, Pavel Hrbacek, Sona Rusnakova, Ondrej Bosak, Marian Kubliha

**Affiliations:** 1 Faculty of Technology, Tomas Bata University in Zlin, Vavreckova 5669, 760 01 Zlin, Czech Republic; straznicky@utb.cz (P.S.); p_hrbacek@utb.cz (P.H.); rusnakova@utb.cz (S.R.); 2 Department of Machining, Assembly and Engineering Metrology, Faculty of Mechanical Engineering, VŠB-Technical University of Ostrava, 17. listopadu 2172/15, Poruba, 708 00 Ostrava, Czech Republic; 3 Faculty of Materials Science and Technology, Slovak University of Technology, Bottova 25, 917 24 Trnava, Slovakia; ondrej.bosak@stuba.sk (O.B.); marian.kubliha@stuba.sk (M.K.)

**Keywords:** sheep wool, polymer composites, mechanical vibration, sound absorption, electrical conductivity, light transmission, Wdls 5.0 software

## Abstract

Sheep farmers are currently facing an oversupply of wool and a lack of willing buyers. Due to low prices, sheep wool is often either dumped, burned, or sent to landfills, which are unsustainable and environmentally unfriendly practices. One potential solution is the utilization of sheep wool fibers in polymer composites. This paper focuses on the study of mechanical vibration damping properties, sound absorption, light transmission, electrical conductivity of epoxy (EP), polyurethane (PU), and polyester (PES) resins, each filled with three different concentrations of sheep wool (i.e., 0%, 3%, and 5% by weight). It can be concluded that the sheep wool content in the polymer composites significantly influenced their physical properties. The impact of light transmission through the tested sheep wool fiber-filled polymer composites on the quality of daylight in a reference room was also mathematically simulated using Wdls 5.0 software.

## 1. Introduction

Sheep wool is a natural animal fiber derived from the shear activity of the fleece of sheep. It consists of 60% animal protein fibers, 15% moisture, 10% fat, 10% sheep sweat, and 5% impurities [1,2]. The leading countries in wool production are Australia, China, the United States, and New Zealand. The global production of greasy wool is approximately 2 million tons from one billion sheep [3]. There are more than 200 different breeds of sheep, each with its own unique type of wool. The most popular breeds for wool production are Merino, Romney, and Blue-faced Leicester [4].

Sheep wool is a fiber of the highest quality, cleanest, and most environmentally friendly insulation materials in the world with excellent properties [5]. Wool fibers, which do not irritate the eyes, skin, or lungs, pose no danger to human health. They are characterized by moisture absorption and desorption, breathability, high durability, good thermal, vibration damping and sound insulation properties, and resistance to fire, static electricity, and dirt [6,7,8,9,10]. Due to these properties, sheep wool is widely used in many areas. Sheep wool is traditionally utilized in the textile industry to produce common woolen products, such as carpets, garments, curtains, covers, and bedding [11]. Coarse wool is also used as a filling material for mattresses and pillows [12]. In addition to the textile industry, sheep wool is now also used in other branches of the economy, such as building, civil engineering, cosmetics, and medical products, as well as in organic farming [13]. However, sheep are reared mainly for their meat and to a lesser extent for their milk, but not for their wool [14]. Therefore, sheep wool is currently considered as a by-product of sheep farming, not only because of its low quality but also because of low prices [15]. It is estimated that at least 200,000 tons of low-quality wool are produced annually in the European Union [14]. As a result, sheep wool is often incinerated, dumped, or sent to landfills, which are unsustainable and environmentally unfriendly practices [15,16]. With a growing interest in the sustainable use of natural resources, wool has been reconsidered as an underrated and underutilized renewable resource deserving of more effective exploitation [17]. Currently, sheep wool can also be used as a filler in composite materials. In general, the use of sheep wool as a component in different composite materials has been investigated, including sheep wool-based cement, polymer and resin composites, and sandwich panels with sound-absorbing and thermal-insulating properties, among others.

The effect of sheep wool fibers on thermal insulation and mechanical properties of cement-based composites was studied by Fiore et al. [18]. In this case, the composites were produced with wool fibers at three different maximum lengths (i.e., 1, 6, and 20 mm) and three different weight percentages (i.e., 13%, 23%, and 46%). The results indicated that wool fibers significantly enhanced thermal insulation and reduced the compressive strength of the composites, compared to the neat cementitious matrix, regardless of the fibers’ content and length. The highest compressive strength was achieved for a cement-based composite containing 6 mm long wool fibers with a weight percentage of 13%. Alyousef et al. [19] investigated the effect of curing time, treatment process (i.e., modification), and weight percentages (i.e., from 0 to 6%) of sheep wool, with a length of 70 mm on the mechanical properties of cement-based composites. They reported that adding sheep wool fibers to concrete mixes led to higher tensile and flexural strength values, while reducing compressive strength. Additionally, it was found that curing time and a saltwater treatment modification of sheep wool enhanced the mechanical properties of the tested concrete specimens. Mourin et al. [20] investigated the impact of incorporating sheep wool into unfired blocks (pure clay, clay with 3% wool, and clay with 5% wool). Their research demonstrated that the composite containing 5% wool and clay exhibited the lowest thermal conductivity and transmittance, indicating superior thermal insulation properties. The effect of sheep wool and hemp fibers on the mechanical properties of gypsum-based composites was studied by Fantilli et al. [21]. They found that the addition of sheep wool and hemp fibers reduced the compressive and flexural strengths by approximately 20% compared to plain gypsum. Conversely, the flexural toughness of the gypsum-wool composites was significantly higher than that of the pure gypsum sample. A multilayer sheep wool-reinforced porous plaster was investigated by Atbir et al. [22]. The results showed that increasing the number of wool layers led to a rise in flexural strength and a reduction in density, compressive strength, and thermal conductivity of the plaster composites studied. Maia Pederneiras et al. [23] analyzed the impact of the incorporation of 10% and 20% (in volume) of 1.5 cm and 3.0 cm wool fibers in cement and cement-lime mortars. The results showed that the incorporation of wool fibers increased the ductility of the mortars and improved their mechanical properties. A positive effect of sheep wool on the mechanical properties of composites composed of hydrated lime, organic admixtures (such as wheat, rice, and corn pastes), and wool fibers, was observed in [24].

An experimental investigation on mechanical and thermal characteristics of waste sheep wool fiber-filled epoxy composites was carried out by Sharma et al. [25]. They fabricated four different types of epoxy composites filled with waste sheep wool, all with the same fiber content by weight but differing in density and void content. The results indicated that these composites, due to their lightweight, reduced thermal conductivity, and enhanced mechanical properties, can be used as insulating building components. Semitekolos et al. [26] investigated the thermal and mechanical properties of wool fiber-toughened epoxy composites with wool contents of 2.4, 4.1, and 5.7 parts per hundred resins (phr). In this case, sheep wool was sourced from two different Greek sheep breeds (i.e., Kalarritiko and Katsika). It was found that small amounts (i.e., up to 4.1 phr) of both types of wool fibers can significantly improve the thermal insulating properties of the wool–fiber epoxy composites, while maintaining the epoxy’s mechanical properties. In a study [27], mechanical properties, water absorption, chemical absorption, and biodegradability tests were conducted on woven sheep wool–fiber reinforced with 40% and 50% epoxy composites. The results showed that increasing the sheep wool content led to an increase in tensile and bending strengths, as well as water absorption, while chemical absorption decreased. The biodegradability of both wool–fiber epoxy composites remained practically the same after a few days. The effect of sodium lignosulfonate (SLS) treatment on wool fabric during the preparation of wool–latex rubber composites was studied in [28]. SLS was used to enhance the surface interaction between the wool fabric and the natural rubber latex. It was found that low SLS concentrations resulted in a marginal increase in tensile strength properties. In addition, the wool–SLS–rubber composites showed favorable UV ageing properties. Sheep wool fibers were used as reinforcement for poly (lactic acid) (PLA) plasticized with maleinized linseed oil, resulting in a decrease in tensile strength but an increase in the modulus of elasticity, hardness, and elongation at break [29]. Magnat et al. [30] compared the biodegradability of pure PLA specimens with those of PLA–silk and PLA–sheep wool composites. They observed that the biodegradation rate of PLA–sheep wool specimens was lower compared to both pure PLA and PLA–silk specimens. The mechanical behaviors of sheep wool fiber reinforced polyester (SHFRP) composites, fabricated using compression molding, and varying fiber weight percentages of 20%, 30%, and 40%, were evaluated in [31]. The study found that the composites with 40 w.% fiber loading exhibited the highest tensile and flexural strengths, whereas the highest tensile modulus was recorded for the 20 w.% SHFRP composite sample. In contrast, the 30 w.% SHFRP composites demonstrated the highest impact strength. The mechanical properties of the hybrid polypropylene composites reinforced with jute and sheep wool fibers were examined by varying the fiber and polypropylene ratio and by varying wool and jute fiber ratio, as detailed in [32]. The results indicated that mechanical properties (i.e., tensile, flexural and impact strength, Young’s modulus of elasticity, flexural modulus, and Shore D hardness) at a jute and wool fiber ratio of 1:1 improved with increasing fiber content. It was also found that the best mechanical properties were obtained from 15% fiber loading with a jute and wool fiber ratio of 3:1.

Sheep wool is further utilized to enhance the sound absorption properties of building materials. Tămaş-Gavrea et al. [33] investigated the sound absorption properties of perforated sheep wool-based sandwich panels with a height of 50 mm and compared them with glass wool (GW) and expanded polystyrene (EPS), each measuring 25 mm in thickness. The study concluded that the wool-based sandwich panels exhibited superior sound absorption across almost all excitation frequencies (from 125 to 2000 Hz) compared to the EPS samples. Conversely, these panels demonstrated higher sound absorption coefficients than the GW samples, only at lower excitation frequencies (from 250 to 500 Hz). Dénes et al. [34] studied the sound absorption properties of polymer–sheep wool composites that were prepared using two types of binders (i.e., acrylic–polyurethane resin and natural rubber latex). The composites were produced with uniform wool concentration across the binders. It was found that the wool–resin composites exhibited a high sound absorption over the entire frequency range (i.e., from 100 to 3200 Hz), while the wool–latex composites were only more efficient at high frequencies. In general, the sound absorption properties of both composites increased with thickness, as evidenced by higher values of the noise reduction coefficient. Furthermore, the wool–resin composites exhibited a higher thermal conductivity and water absorption compared to the wool–latex composites. The sound absorption properties of sheep wool–soy protein biocomposites were investigated by Urdanpilleta et al. [35]. This study revealed that these biocomposites exhibited good acoustic absorption performance across a broad frequency range, from 100 to 5000 Hz, comparable to conventional acoustic absorbers such as glass wool and polyurethane foams. Therefore, these findings suggest that synthetic materials can be replaced by natural ones to improve the sustainability of the building sector.

The effect of a stacking sequence on the physical and chemical properties of a hybrid composite, consisting of biomass sheep wool fiber and glass fiber reinforced with epoxy, was investigated in [36]. In this study, three composites were produced, each consisting of 40% epoxy, 30% biomass woven sheep wool fiber, and 30% glass fabric. The reinforcements (i.e., two layers of wool fibers and twenty-one layers of glass fibers) were placed in the required sequence between two layers of the epoxy matrix. The findings indicated that the composite with sheep wool layers placed directly on the epoxy matrices (i.e., with glass fibers in the middle) demonstrated superior tensile and bending strength and exhibited a higher moisture and chemical absorption compared to the other two composites. Other studies have shown that sheep wool can also be used in sheep wool-based composites as a filtration material [37] for cleaning various fluids and as a suitable fibrous component in multilayer nonwoven fabrics for clothing and footwear [38].

As mentioned above, many researchers have investigated various properties of sheep wool-based composites, including mechanical properties, thermal resistance, flammability, and moisture resistance. However, this work did not investigate these physical properties. This study extends the research to other physical properties of resin–sheep wool composites. The aim of this work is to investigate the effect of sheep wool in three different (i.e., epoxy, polyurethane, and polyester) resins on mechanical vibration damping properties, sound absorption, light transmission, and electrical conductivity of these polymer–sheep wool composites. In this case, the investigated polymer specimens were filled with three different concentrations of sheep wool (i.e., 0%, 3%, and 5% by weight). To the best of the author’s knowledge, no relevant studies have yet been published investigating the mentioned physical properties of resin–sheep wool composites. The results presented in this paper could help in the development of the polymer–sheep wool composites for practical applications, such as light and electrical insulation materials, thereby benefiting our environment.

## 2. Materials and Methods

### 2.1. Production of Polymer Composite Samples

**Figure 1:**
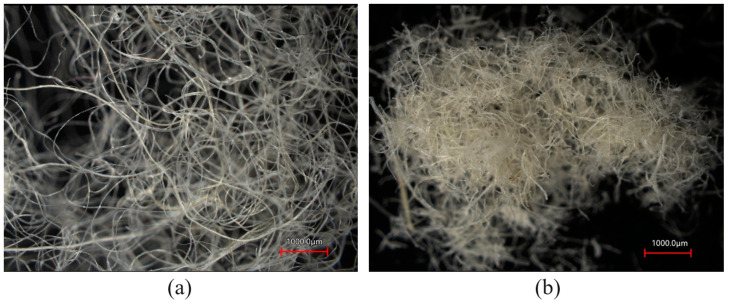
Microscopic structures of sheep wool fibers: (**a**) before milling process; (**b**) after milling process.

A Naturwool A500/P100 sheep fleece insulation strip from Naturwool Ltd. (Prague, Czech Republic) was used as the primary material for producing the polymer–sheep wool composites. Some properties of the sheep fleece strip are given in Table 1. Initially, the sheep fleece insulation strip was cut into smaller parts using scissors. Then, sheep fibers were milled by passing them through mesh sizes of 3 mm and 1 mm, using the universal cutting mill PULVERISETT 19 (FRITSCH GmbH-Milling and Sizing, Weimar, Germany). The structure of the sheep fibers before and after the milling process was evaluated using a VK-X3000 laser scanning microscope (LSM, Keyence Corp., Osaka, Japan), as shown in Figure 1. It was found that the fiber diameter was in the range *d* = (15.9 ÷ 39.5) μm. The sheep wool processed in this way was subsequently used to produce polymer–sheep wool composite materials.

**Figure 2:**
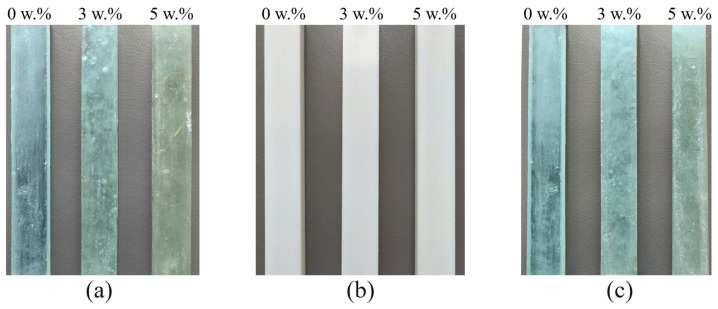
Photographs of the investigated polymer samples with different concentrations of sheep wool *W_r_* (in w.%): (**a**) EP samples; (**b**) PU samples; (**c**) PES samples.

A total of three different resins, namely epoxy (EP), polyurethane (PU), and polyester (PES) resins, were filled with three different weight concentrations (i.e., 0 w.%, 3 w.%, and 5 w.%) of sheep wool in this study. The designation of the used resins are given in Table 2. These resins were produced by Dawex Chemical Ltd. (Prague, Czech Republic). The test samples were created using a mold made of Lukopren N 1522 silicone rubber (Lucebni zavody JSC, Kolin, Czech Republic) type at an ambient temperature of 22 °C. The curing process involved adding the appropriate hardener to the resin, according to the manufacturer’s recommendations. The manufacturing process involved separating the mold using a V11 separator (Dawex Chemical Ltd., Prague, Czech Republic) and applying the given resin with the corresponding weight ratio of sheep fibers. The orientation of the filler in the polymer matrices was random and performed manually. The test specimens were not further cured due to temperature influence. The formation of cross-linked structures was observed in the studied samples during the curing process, with the sheep wool-based filler well dispersed in the polymer matrix. Photographs of the tested polymer samples with different concentrations of sheep wool are shown in Figure 2.

### 2.2. Measurement Methodology

#### 2.2.1. Vibration Damping Testing

The dynamic mechanical properties of the studied polymer materials were evaluated based on the displacement transmissibility *T_d_* (–), which is defined as [39,40]:

(1) $$T_{d}=\frac{x_{O}}{x_{I}}=\sqrt{\frac{k^{2}+{\left(c\omega \right)}^{2}}{{\left(k-m\omega^{2}\right)}^{2}+{\left(c\omega \right)}^{2}}}=\sqrt{\frac{1+{\left(2\zeta r\right)}^{2}}{{\left(1-r^{2}\right)}^{2}+{\left(2\zeta r\right)}^{2}}}$$

where *x* is the displacement amplitude on either the input (*I*) or output (*O*) sides of the tested sample, *k* is the sample stiffness (N/m), *c* is the viscous damping coefficient (N·s/m), *ω* is the circular frequency of oscillation (rad/s), *m* is the mass (kg), *ζ* is the damping ratio (–), and *r* is the frequency ratio (–).

In general, depending on the displacement transmissibility value, there are three different types of mechanical vibrations, namely damped (*T_d_* < 1), undamped (*T_d_* = 1), and resonance (*T_d_* > 1) mechanical vibrations. Under the condition *dT_d_*/*dζ* = 0 in Equation (1), is it possible to determine the frequency ratio *r*_0_ at which the displacement transmissibility reaches its local extremum (i.e., its maximum value *T_dmax_*) at the first resonance frequency *f_R_*_1_ [39,41]:

(2) $$r_{0}=\frac{\sqrt{\sqrt{1+8\zeta^{2}}-1}}{2\zeta }$$

It is evident from Equation (2) that the maximum value of the displacement transmissibility is generally shifted to lower values of the frequency ratio *r* with increasing damping ratio *ζ* (or decreasing mechanical stiffness *k*) [42,43].

**Figure 3:**
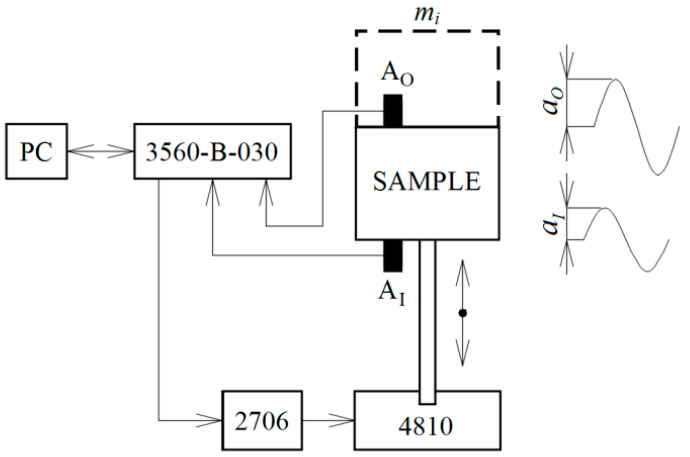
The schematic diagram of the experimental apparatus for measuring the displacement transmissibility of a linear single-degree-of-freedom system.

Experimental measurements of the displacement transmissibility of the tested sheep wool-filled polymer composites were performed using the method of harmonically excited mechanical vibrations within the frequency range of 2–1000 Hz. In addition, the vibration damping properties of the natural sheep wool fibers were evaluated in the frequency range from 2 to 200 Hz. The schematic diagram of the measuring apparatus (Brüel & Kjær, Nærum, Denmark) for evaluating the vibration damping properties of a linear single-degree-of-freedom (SDOF) system is shown in Figure 3. The measuring apparatus included a mini-shaker (BK 4810), a signal PULSE multi-analyzer (BK 3560-B-030), and a power amplifier (BK 2706). In the case of the harmonically excited mechanical vibrations, Equation (2) can also be expressed as follows:

(3) $$T_{d}=\frac{a_{O}}{a_{I}}$$

where *a* is the acceleration amplitude on either the input (*I*) or output (*O*) sides of the tested sample. The displacement transmissibility was determined from Equation (3) based on the measured acceleration amplitudes that were recorded using the BK 4393 piezoelectric accelerometers *A_I_* and *A_O_* (Brüel & Kjær, Nærum, Denmark). Frequency dependencies of the displacement transmissibility were determined for the investigated polymer and sheep wool samples, each with ground plane dimensions of 60 mm × 60 mm. The polymer samples under investigation, each with a thickness of 4 mm, were loaded with an inertial mass (*m_i_*) of 90 g, which was placed on top of the tested, harmonically loaded samples, as depicted in Figure 3. Similarly, the sheep wool samples of three different thicknesses (i.e., 10, 20, and 30 mm) were loaded with an inertial mass of 10 g. Each measurement was repeated 5× under ambient conditions at a temperature of 22 °C.

#### 2.2.2. Sound Absorption Properties

The material’s ability to absorb sound is expressed by the sound absorption coefficient *α* (–), defined as the ratio [44]:

(4) $$\alpha =\frac{P_{d}}{P_{i}}$$

where *P_d_* (W) is the dissipated acoustic power, and *P_i_* (W) is the incident acoustic power. The sound absorption properties of a material are affected by several factors, including the frequency of acoustic waves, material thickness, density, structure, and temperature.

**Figure 4:**
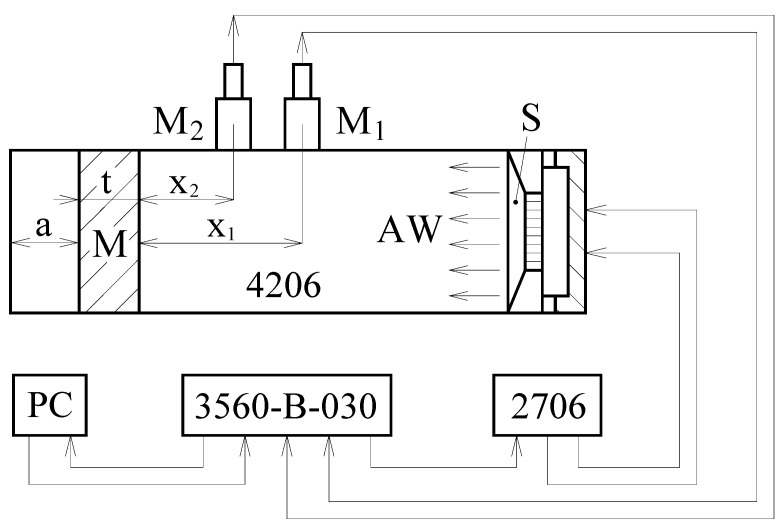
The schematic of the experimental apparatus for measuring frequency dependencies of the sound absorption coefficient. Legend of the abbreviations: *a*—air gap behind tested material sample, AW—acoustic wave, M—measured sample, M_1_ and M_2_—measuring microphones, S—sound source, *t*—sample thickness, *x*_1_ and *x*_2_—distances from the tested material sample to the microphones.

Frequency dependencies of the normal incidence sound absorption coefficient *α* of the tested polymer and sheep wool specimens were determined experimentally using a two-microphone acoustic impedance tube (BK 4206) in conjunction with a power amplifier (BK 2706) and a signal PULSE multi-analyzer (BK 3560-B-030) in the frequency range from 200 to 6400 Hz (Brüel & Kjær, Nærum, Denmark). Figure 4 displays a schematic diagram of the experimental apparatus. The sound absorption properties were evaluated for the same specimen thicknesses as those used in the vibration damping tests. Therefore, the polymer material samples had a thickness of 4 mm, while the thickness of the sheep wool samples ranged from 10 to 30 mm. Additionally, the sound absorption properties of the tested polymer samples were experimentally measured with different air gap sizes *a* (ranging from 0 to 3 cm) behind the tested samples, as shown in Figure 4. All samples were cylindrical in shape with an outer diameter of 29 mm. All measurements were performed at an ambient temperature of 22 °C.

The frequency dependencies of the sound absorption coefficient for the investigated specimens were determined using the two-microphone transfer function method according to ISO 10534-2 [45] standard, based on the partial standing wave principle. Using this method, the normal incidence sound absorption coefficient *α* is defined by the formula [46,47]:

(5) $$\alpha =1-r^{2}$$

where *r* (–) is the normal incidence reflection factor, given by the equation:

(6) $$r=\frac{H_{12}-H_{I}}{H_{R}-H_{12}}e^{2jk_{0}x_{1}}$$

where *H*_12_ (–) is the pressure transfer function, *H_I_* (–) is the transfer function for the incident acoustic wave, *H_R_* (–) is the transfer function for the reflected acoustic wave, *k*_0_ (m^−1^) is the complex wave number, and *x*_1_ (m) is the distance between the microphone M_1_ and the tested material sample (see Figure 4). The transfer functions are expressed as follows:

(7) $$H_{12}=\frac{p_{2}}{p_{1}}=\frac{e^{jk_{0}x_{2}}+r\cdot e^{-jk_{0}x_{2}}}{e^{jk_{0}x_{1}}+r\cdot e^{-jk_{0}x_{1}}}$$

(8) $$H_{I}=e^{-jk_{0}\left(x_{1}-x_{2}\right)}$$

(9) $$H_{R}=e^{jk_{0}\left(x_{1}-x_{2}\right)}$$

where *p*_1_ (Pa) and *p*_2_ (Pa) are the complex acoustic pressures at the positions of the microphones M_1_ and M_2_, and *x*_2_ (m) is the distance between the microphone M_2_ and the tested material sample.

#### 2.2.3. DC Electrical Conductivity Testing

Electrical conductivity *σ* (S·m^−1^) expresses a material’s ability to conduct an electrical current [48] and was determined using the formula [49]:

(10) $$\sigma =\frac{1}{R}\cdot \frac{l}{A}$$

where *R* (Ω) is the electrical resistance, *l* (m) is the distance between electrodes, and *A* (m^2^) is the electrode area.

In this study, the determination of the studied electrical properties of polymer–sheep wool composites was performed with DC electrical conductivity measurements using Novocontrol Concept 90 (NOVOCONTROL Technologies GmbH & Co. KG, Montabaur, Germany). The electrical resistance was determined by measuring the electric current passing through the sample at a constant voltage of 10 V. The electric current was measured by the Keithley 6517B picoammeter (Keithley Instruments LLC., Cleveland, OH, USA).

The samples used for measuring the electrical properties were prepared as thin blocks with dimensions of 15 mm × 15 mm × 2 mm (length × width × thickness). To ensure a sufficiently homogeneous electric field in the tested specimen, a graphite-based electrode was applied to the sample surface. The DC conductivity of the investigated polymer samples was measured in the temperature range from 20 to 80 °C at a constant heating rate of 1 °C/min. Furthermore, in order to mitigate the effects of low molecular weight component release and moisture entry into the measured sample, it was necessary to perform repeated electrical conductivity measurements. It was found that these effects were eliminated in the third measurements. Therefore, the temperature dependencies of the DC conductivity were evaluated after the third heating of the tested polymer samples.

#### 2.2.4. Light Transmission Properties

The material’s ability to transmit light is characterized by the light transmission coefficient *τ* (–), defined by the ratio [50]:

(11) $$\tau =\frac{\Phi_{T}}{\Phi_{I}}$$

where *Φ_T_* (W) is the total transmitted luminous flux, and *Φ_I_* (W) is the incident luminous flux. The light transmission coefficient *τ* was determined in accordance with ČSN 360011-2 [51] using the illuminance ratio method, as given by:

(12) $$\tau =\frac{E_{T}}{E_{I}}$$

where *E_T_* (lx) is the illuminance measured behind the inserted polymer sample, and *E_I_* (lx) is the incident illuminance measured without the inserted polymer sample (i.e., after its removal). Experimental measurements of the light transmission properties of the tested polymer composites were performed during the propagation of diffused daylight through the samples, using a Voltcraft MS-1300 luxmeter (Voltcraft, Hirschau, Germany). To ensure the highest accuracy in determining the light transmission coefficient, the measurements were performed in the shade under clear skies during the summer months, around noon. Since the PU specimens are not transparent, only the EP and PES specimens were experimentally evaluated in terms of light transmittance. The tested block article had dimensions of 60 mm × 60 mm × 4 mm (length × width × thickness). Each measurement was repeated 20× at an ambient temperature of (25 ± 2) °C. Subsequently, mean values and standard deviations of the light transmission coefficient were calculated.

The effect of light transmission through the tested transparent polymer materials on daylight quality was evaluated using the daylight factor *DF* (%), defined by the formula [52]:

(13) $$DF=\frac{E_{x}}{E_{H}}\cdot 100$$

where *E_x_* (lx) is the indoor illuminance measured in a point *x* on the tested working surface, and *E_H_* (lx) is the outdoor diffuse illuminance measured on a horizontal surface. Mathematical simulations of the daylight factor were performed using Wdls 5.0 software (Astra MS Software Ltd., Otrokovice, Czech Republic) based on multiple light reflections in a reference room.

## 3. Results and Discussion

### 3.1. Vibration Damping Properties

**Figure 5:**
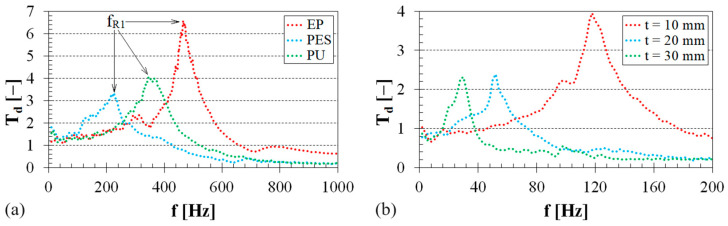
Frequency dependencies of the displacement transmissibility: (**a**) virgin polymer materials, sheep wool concentration *W_r_* = 0 w.%, inertial mass *m_i_* = 90 g; (**b**) pure sheep wool, inertial mass *m_i_* = 10 g.

**Figure 6:**
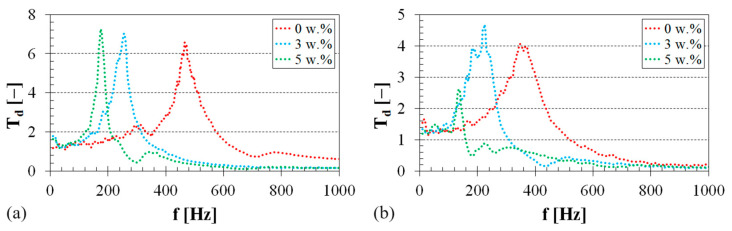
Effect of the sheep wool concentration on the displacement transmissibility: (**a**) EP–sheep wool composites, inertial mass *m_i_* = 90 g; (**b**) PU–sheep wool composites, inertial mass *m_i_* = 90 g.

The frequency dependencies of the displacement transmissibility *T_d_* are depicted in Figure 5 and Figure 6. Figure 5a compares the mechanical vibration damping properties of the three virgin polymer materials, i.e., without sheep wool content. It is evident that the material’s ability to damp mechanical vibrations (i.e., *T_d_* < 1) is generally achieved at higher excitation frequencies. Lower vibration damping properties were observed for the tested EP specimen, which effectively damped mechanical vibrations at frequencies *f* > 650 Hz. Contrarily, the PES specimen exhibited the ability to damp mechanical vibrations at lower excitation frequencies (i.e., at *f* > 420 Hz). This is reflected in the first resonance frequency (*f_R_*_1_) peak position, which generally increases with the increasing sample stiffness *k* (or the decreasing damping ratio *ζ*), as given in Equation (2). The mean values and corresponding standard deviations of the first resonance frequency for the tested polymer specimens are given in Table 3. The effect of the thickness of pure sheep wool, loaded with an inertial mass *m_i_* = 10 g, on the displacement transmissibility is shown in Figure 5b. It is evident that the vibration damping properties of sheep wool generally increased with increasing wool thickness *t*, as indicated by a shift in the first resonance frequency peak position to lower excitation frequencies (see Table 4).

The effect of sheep wool content on the displacement transmissibility of the tested EP/sheep wool and PU–sheep wool composites is shown in Figure 6. It is clear that the vibration damping ability of these polymer composites generally increased with increasing sheep wool content. This is attributed to the significantly lower mechanical stiffness of sheep wool (as depicted in Figure 5) compared to the mechanical stiffness of the virgin polymers (i.e., *W_r_* = 0 w.%). Consequently, the increase in sheep wool content led to a shift in the first resonance frequency peak position towards lower excitation frequencies, as demonstrated in Figure 6a (for EP–sheep wool composites) and Figure 6b (for PU–sheep wool composites). A similar effect was also observed in the examined PES–sheep wool composites (see Table 3). For the reasons mentioned above, increasing the sheep wool content in the polymer matrix generally led to a higher conversion of mechanical energy into heat under harmonic dynamic loading of the investigated polymer composites.

### 3.2. Sound Absorption Properties

**Figure 7:**
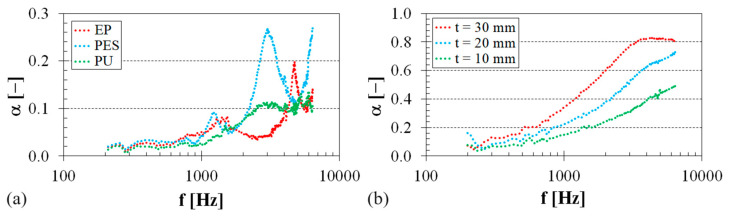
Frequency dependencies of the sound absorption coefficient: (**a**) virgin polymer materials, air gap size *a* = 0 mm; (**b**) pure sheep wool, air gap size *a* = 0 mm.

The frequency dependencies of the sound absorption coefficient for both the virgin polymers and pure sheep wool under investigation are depicted in Figure 7. It is evident that the PES specimen exhibited a higher ability to damp sound compared to the other examined polymer specimens. This effect is primarily observed at higher excitation frequencies (i.e., at *f* > 2 kHz), as shown in Figure 7a. The lowest sound damping properties were observed for the investigated EP sample, particularly in the frequency range from 1500 to 4000 Hz. These findings are in excellent agreement with the results of vibration damping tests, indicating that the investigated virgin EP–PES samples exhibited the highest–lowest mechanical stiffness under harmonic dynamic loading. Therefore, lower material mechanical stiffness was associated with improved sound damping properties in the studied polymer specimens. It was also observed (see Figure 7a) that the ability of the investigated polymer samples to absorb sound was very similar at lower excitation frequencies (i.e., at *f* < 700 Hz). However, the sound absorption properties of the studied polymers were generally low across the entire frequency range. The highest value of the sound absorption coefficient (i.e., *α_max_* = 0.268) was recorded for the virgin PES specimen at the excitation frequency of *f* ≅ 3 kHz. For this reason, it can be concluded that the studied virgin polymer materials are not suitable for sound absorption. In contrast, pure sheep wool exhibited a higher capability for noise damping compared to the virgin polymer specimens.

The frequency dependencies of the sound absorption coefficient of pure sheep wool for three different wool thicknesses are shown in Figure 7b. It is obvious that the sound absorption properties of sheep wool increased with the excitation frequency and the sample thickness. The maximum value of the sound absorption coefficient (i.e., *α_max_* = 0.836) was found at the excitation frequency of *f* = 4264 Hz for a 30 mm thick sample. The better sound damping properties of sheep wool compared to the investigated virgin polymer samples are due to the structure of the wool fibers because porous, fiber, and spongy structures are generally used as sound absorbers [53]. In this case, the incident acoustic energy is primarily dissipated as heat due to the friction between air molecules and the pore walls, along with viscous losses resulting from airflow viscosity within the materials [54]. For the reasons mentioned above, the sound absorption properties of the investigated virgin polymers can be improved by adding sheep wool fibers into their matrix.

**Figure 8:**
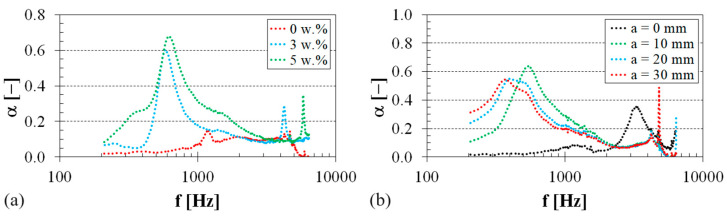
Frequency dependencies of the sound absorption coefficient: (**a**) PU–sheep wool composite, air gap size *a* = 10 mm; (**b**) PES–sheep wool composite, sheep wool concentration *W_r_* = 3 w.%.

The impact of sheep wool content on the sound absorption performance of the tested PU–sheep wool composites is depicted in Figure 8a. In this case, the air gap size behind these composites was 10 mm. It is evident that the addition of sheep wool fiber to the virgin PU polymer improved the sound absorption properties of these composite specimens, particularly at lower excitation frequencies (i.e., at *f* < 1 kHz). The maximum value of the sound absorption coefficient (i.e., *α_max_* = 0.679) was found at the excitation frequency of *f* = 612 Hz for the PU–sheep wool composite with a sheep wool concentration of *W_r_* = 5 w.%. Thus, increasing the sheep wool concentration generally improved the sound damping properties of the tested PU–sheep wool composites, accompanied by a greater conversion of acoustic energy into heat. Similar conclusions were also found for the other (i.e., EP–sheep wool and PES–sheep wool) composites examined.

The air gap size *a* (see Figure 4) behind the tested polymer sample also significantly affects its ability to absorb sound. Figure 8b shows the effect of the air gap size on the sound absorption properties of the PES–sheep wool composite with a sheep wool concentration of *W_r_* = 3 w.%. It is obvious that the air gap size generally led to improved sound absorption properties of this composite material. It is also visible that the primary sound absorption maximum of the sound absorption coefficient *α_max1_* was shifted towards lower excitation frequencies with the increasing air gap size *a*. Therefore, adjusting the air gap size behind the tested polymer sample can be used to improve the sound insulation properties, especially at low excitation frequencies. This phenomenon is characteristic of open-porous material structures, where the maxima of the sound absorption coefficient are proportional to odd multiples of quarter wavelengths at standing-wave antinodes and corresponding excitation frequencies [55,56].

### 3.3. Electrical Conductivity

**Figure 9:**
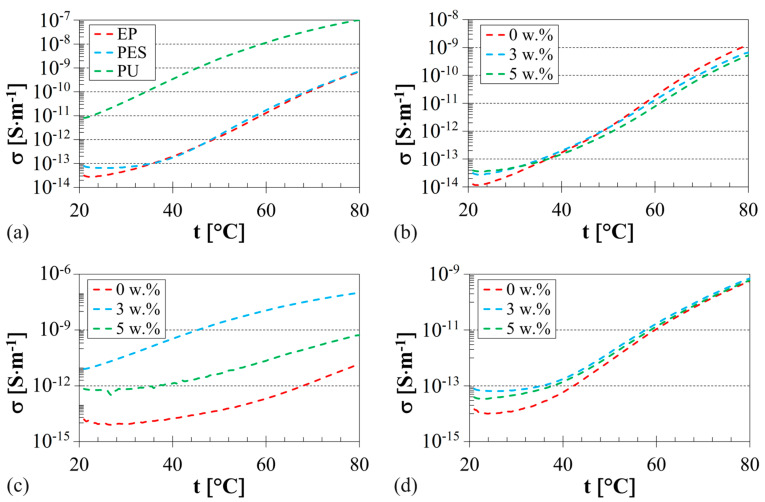
Temperature dependencies of the DC electrical conductivity: (**a**) polymer–sheep wool composites, sheep wool concentration *W_r_* = 3 w.%; (**b**) EP–sheep wool composites; (**c**) PU–sheep wool composites; (**d**) PES–sheep wool composites.

The temperature dependencies of the DC electrical conductivity for the investigated polymer composites are shown in Figure 9. It is evident that the DC electrical conductivity generally increased with increasing temperature, which correlates with the deteriorating electrical insulating properties of the polymer composites.

It was observed that the addition of sheep wool fibers to the virgin EP and PES polymer matrix had no significant effect on their DC conductivity, as depicted in Figure 9b,d. The DC conductivity of the studied EP–sheep wool and PES–sheep wool composites remain very similar, regardless of the sheep wool concentration (see Figure 9a). A more pronounced impact on the DC conductivity of these composites is attributed to the moisture absorption–desorption effect. This phenomenon was observed for the PES–sheep wool composite at the temperatures *t* < 50 °C (see Figure 9d). In the case of the EP–sheep wool composite, the moisture absorption–desorption effect was evident over the whole measured temperature range, especially at the highest sheep wool concentration (i.e., *W_r_* = 5 w.%). Consequently, the addition of sheep wool fiber to the EP–sheep wool composite enhanced the moisture desorption effect, making it suitable for power electronics applications. In cases where equipment operates under the influence of heat, i.e., at higher operating temperatures, the DC electrical conductivity is reduced. This reduction enhances the electrical insulating ability of these composites, as depicted in Figure 9b.

Compared to the studied EP–sheep wool and PES–sheep wool composites, it was observed that the sheep wool concentration in the PU–sheep wool composite had a significant effect on the DC electrical conductivity, as depicted in Figure 9c. At first, the DC conductivity significantly increased in the PU–sheep wool composite with a sheep wool concentration of 3 w.%, leading to a deterioration of the electrical insulating properties of this composite. Subsequently, at the highest concentration (i.e., *W_r_* = 5 w.%) of sheep wool in the PU–sheep wool composite, the DC electrical conductivity decreased, resulting in the improved electrical insulating properties of these composites. Therefore, sheep wool fibers at concentrations *W_r_* < 3 w.% act as an antistatic additive in the PU–sheep wool composites.

### 3.4. Light Transmission

As mentioned above, the light transmission properties were experimentally determined only for the EP–sheep wool and PES–sheep wool composites due to the opacity of the PU specimens (see Figure 2b). The experimentally measured values of the light transmission coefficient *τ*, including their standard deviations, are given in Table 5 for three different values of the sheep wool concentration *W_r_*. These values were determined according to Equation (12) based on the experimentally measured illuminances E_T_ and E_I_.

It can be concluded that the light transmission properties of the tested polymer composites significantly decreased with the increasing concentration of sheep wool. In the case of the highest concentration of sheep wool (i.e., *W_r_* = 5 w.%), the light transmission coefficient decreased from 26% to 46% for the PES–sheep wool and EP–sheep wool composites compared to the virgin PES and EP samples, respectively.

**Figure 10:**
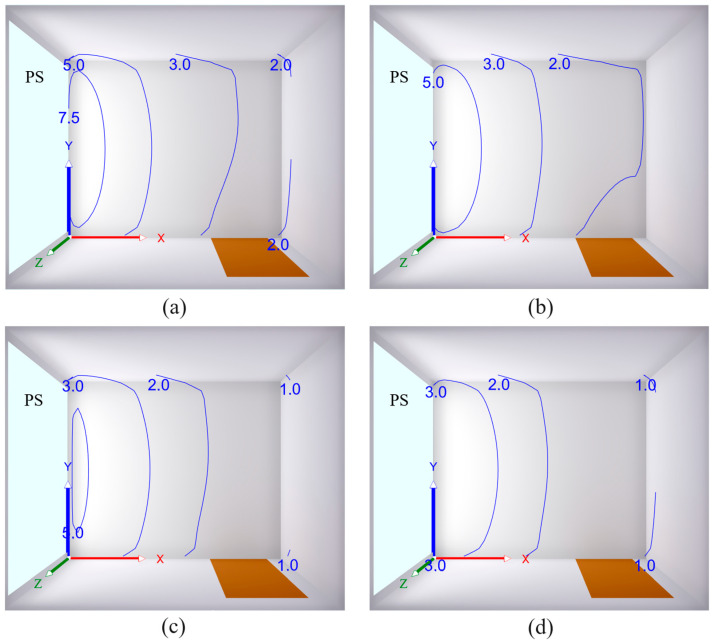
Distribution of the daylight factor *DF* (in %) at a height of 0.75 m above the room floor depending on the light transmission coefficient *τ* of the tested polymer specimens (PS): (**a**) *τ* = 0.72; (**b**) *τ* = 0.58; (**c**) *τ* = 0.43; (**d**) *τ* = 0.37.

The effect of the sheep wool concentration on daylight quality was evaluated by mathematical simulations of the daylight factor using Wdls 5.0 software. These simulations were based on multiple light reflections in a reference room with dimensions of 3.0 m × 2.5 m × 2.4 m (length × width × height), as shown in Figure 10. The tested polymer sample PS, depicted in blue, had dimensions of 2300 mm × 2300 mm × 4 mm (length × height × thickness) and was embedded in the left wall of the reference room (see Figure 10). In this case, the daylight factor isolines were plotted on the XY plane, located 0.75 m above the room’s floor. The distribution of the daylight factor in the room is significantly influenced not only by the light transmission coefficient *τ* of the tested polymer samples, but also by the light reflection properties of the rooms surfaces, characterized by the light reflectance *ρ* (–) [57]:

(14) $$\rho =\frac{\Phi_{R}}{\Phi_{I}}$$

where Φ*_R_* (W) is the reflected luminous flux from a given surface area. The light reflectance values for the individual surfaces in the reference room are given in Table 6.

The mathematical simulations of daylighting clearly show that the PES sample without sheep wool exhibited the highest values of the daylight factor *DF* (see Figure 10a) and therefore the best daylight quality in the reference room. This sample is characterized by the maximum value of the light transmission coefficient (i.e., *τ* = 0.72). Furthermore, it is apparent that the daylight factor values at a given point generally decreased with a decrease in the light transmission coefficient or an increase in the concentration of sheep wool in the tested polymer–sheep wool composites (see Figure 10b–d). The worst daylighting conditions were observed for the EP–sheep wool composite with a sheep wool concentration of 5 w.%, which is characterized by a minimum value of the light transmission coefficient (i.e., *τ* = 0.37), as depicted in Figure 10d.

The above findings are consistent with the minimum (*DF_min_*), mean (*DF_m_*), and maximum (*DF_max_*) values of the daylight factor obtained using mathematical simulations and are summarized in Table 7 as a function of the sheep wool concentration *W_r_* in the studied polymer specimens.

### 3.5. Comparison and Application of Natural Fibers Composites

Based on their origin, natural fibers (NFs) are classified into three major groups [58]:

- AFs: Animal fibers, derived from animal hair or secretions;
- VFs: Vegetal (plant) fibers, derived from various plant parts;
- MFs: Mineral fibers, derived from inorganic natural resources.

The physical and mechanical properties of the various types of natural fibers, which have potential for composite applications, are presented in Table 8. It is evident that animal fibers, including sheep wool, exhibit lower mechanical properties, such as tensile strength and Young’s modulus of elasticity, compared to vegetal and mineral fibers. It should be noted that the mechanical properties of sheep wool are also affected by the conditioning process. Starkova et al. [59] reported that the long-term conditioning of fibers in a humid environment and under UV irradiation resulted in a significant decrease in tensile strength and elastic modulus. In contrast (see Table 8), the elongation at break values for sheep wool and silk are significantly higher than those of other natural fibers. Furthermore, sheep wool is distinguished by its very high water absorption capacity [60,61,62], reaching up to 35%, compared to other natural fibers such as cotton (up to 25%), jute (12%), hemp (8%), and basalt (less than 1%).

Natural fibers can also be incorporated into polymer composites to modify various properties of virgin polymers such as epoxy resin, polyester, polylactic acid, polyurethane, vinyl ester, and polystyrene. In general, the incorporation of natural fibers into polymers aims to create composite materials that are environmentally friendly, cost effective, with reduced density, and mechanically robust, with specific properties tailored to various applications [63,64].

Composites made of polymer and vegetal fibers are extensively utilized across a range of industries. Polymer composites reinforced with flax, banana, sisal, kenaf, hemp, and jute are utilized in various automotive components, such as headliners, seat back panels, door fosters, side and back walls, seat backs, pillars, and door panels [64]. Muthalagu et al. [65] discovered that reinforcing epoxy composites with Kevlar and date palm fibers enhances their tensile properties. Consequently, these epoxy composites can serve as viable replacements for existing automobile bumper beams made from Glass Mat Thermoplastic (GMT) material. Coconut fibers are used in seat backrests, headrests, interior trims, seat cushioning, and seat bottoms. Cotton fibers play an important role in improving insulation and soundproofing properties. The geopolymer matrix can be reinforced with natural fibers, such as bamboo, flax, hemp, and jute, offering additional benefits. These include enhanced tensile and flexural strength, reduced density, and improved thermal and acoustic insulation properties. Plant fiber-reinforced polymers have a range of potential applications [63], including bamboo composite door panels, roofs incorporating jute coir composites, bicycle frames made from flax–fiber composites, tables featuring oil palm-based biocomposites, perfume containers utilizing Curaua fiber–wood–flour composites, acoustic absorbers composed of cotton fiber–rubber granulate composites, building panels constructed from sisal jute sandwich composites, chairs made of coir fiber–polyester composites, tennis rackets made from hemp epoxy composites and flax, fire retardant materials, ballistic applications, food packaging, etc.

Animal fibers are the second most widely utilized fibers for composite reinforcement, following plant-based fibers [63]. They are characterized by thermal stability and fire resistance. Due to their chemical composition, animal fibers interact well with polymer matrices and increase tolerance to alkaline environments. Additionally, these fibers are non-toxic, eco-friendly, contribute to the creation of biodegradable composites, and exhibit cross-linking properties. In addition, they offer a better moisture absorption capacity compared to plant fibers. However, animal fibers are more expensive compared to plant fibers. Silk derived from silkworms is utilized in tissue engineering as a biomaterial for repairing ligaments, tendons, and bones and in the automotive industry as reinforcement in composite interior parts for commercial and passenger vehicles. Chicken feather fiber (CFF) composites are notable for their robustness, water resistance, low bulk density, and excellent thermal and acoustic insulation properties. As a result, CFF composites find widespread use across various domains. In architecture and civil engineering, they enhance insulation and structural properties in wall panels and roofs. In the transport industry, they contribute to automotive inner insulation parts and aircraft body components, reducing weight and improving efficiency. CFF composites also have applications in the biomedical field, including hydrogels, scaffolds, hydrofilms, and implants, and in the electrical sector, where they are used in printed circuit boards, electrical insulators, and sensors. In addition, they are utilized in food packaging, thermal insulation, filtration, and fireproofing applications. Human hair, due to its considerable strength, can serve as sutures in most surgical procedures. Sheep wool fibers exhibit a rough surface along their entire length, enhancing interfacial adhesion between the fiber and polymer matrix as well as mechanical properties [58]. Consequently, these fibers are utilized as reinforcement in polymer materials. Due to the abovementioned unique properties of sheep wool, polymer composites based on sheep wool find applications across multiple industries [4]. In the automotive industry, they are used in bumpers, dashboards, and door panels. The construction industry benefits from them in lightweight and fire-resistant building materials, such as insulation, roofing, and wall panels. Furthermore, they are utilized in the production of furniture, including chairs and tabletops, and in aerospace for the production of aircraft interiors and structural components. These composites also have medical applications, such as in the production of orthopedic implants, and are used to create lightweight and durable sports equipment, including ski poles, tennis rackets, and bicycle frames. Additionally, they contribute to the production of eco-friendly packaging materials.

It is evident that natural fiber polymer composites, including those made with sheep wool, are widely utilized across various fields due to their properties. The future development of these composites appears promising for several reasons, such as environmental sustainability, reduced carbon footprint, cost effectiveness, weight reduction, growing consumer demand, and enhanced physical and mechanical properties.

## 4. Conclusions

The aim of this work was to investigate the effect of sheep wool content on some physical properties of three different polymer materials, namely epoxy, polyurethane, and polyester resins. In this study, the virgin polymer materials were filled with three different concentrations of sheep wool (i.e., 0%, 3%, and 5% by weight).

It can be concluded that the sheep wool content in the investigated polymer–sheep wool composites significantly influenced their physical properties. Based on the non-destructive method of forced oscillations, it was found that the increased concentration of sheep wool in polymer–sheep wool composites reduced their mechanical stiffness. The decreasing mechanical stiffness resulted in a shift in the first resonance frequency peak position towards lower excitation frequencies. Furthermore, the increased concentration of sheep wool in the polymer–sheep wool composites led to enhanced sound absorption properties, which was attributed to the higher conversion of acoustic energy into heat in the investigated polymer composites. This phenomenon was observed mainly at lower excitation frequencies, especially at *f* < 1 kHz. The impact of sheep wool content on DC electrical conductivity was found only in the polyurethane–sheep wool composites, where the electrical conductivity significantly increased compared to the virgin polyurethane resin. Higher electrical conductivity generally resulted in lower electrical insulating properties of these composites. The polyurethane–sheep wool composite with a 3 w.% wool content exhibited the highest electrical conductivity. In contrast, the effect of sheep wool on the DC electrical conductivity in the epoxy–sheep wool and polyester–sheep wool composites was negligible. This study also found that sheep wool fibers significantly reduced the light transmission properties of the tested transparent epoxy–sheep wool and polyester–sheep wool composites. The epoxy–sheep wool composite with a 5 w.% wool content exhibited the most substantial decrease in light transmission, approximately 46%, compared to the virgin epoxy resin. The negative effect of sheep wool content in the studied polymer composites on daylight quality was also confirmed by mathematical simulations of the daylight factor in a reference room using Wdls 5.0 software.

Currently, sheep farmers are faced with a surplus of sheep wool and a lack of buyers, leading to common practices of discarding, burning, or sending it to landfills. However, these practices are unsustainable and environmentally unfriendly. One possible solution involves integrating sheep’s wool into polymer materials. It can be concluded that the investigated polymer–sheep wool composites are suitable for various applications where high mechanical stiffness are not required. Examples of such applications include sound and light insulation materials, interior design elements, and mechanical vibration dampers at higher excitation frequencies. In certain cases, these composites can be used as electrical insulation materials for power electronics applications at higher operating temperatures. Polymer composites reinforced with sheep wool can also be used as lightweight, fire-resistant, eco-friendly, and biodegradable materials. In addition, they provide further benefits, including water and chemical absorption, thermal insulation, and enhanced mechanical properties.

## Figures and Tables

**Table 1:** Properties of the Naturwool A500/P100 sheep fleece insulation strip.

Parameter	Value
Thickness (mm)	50 *
Mass per unit area (g/m^2^)	500 *
Thermal conductivity coefficient (W·m^−1^·K^−1^)	0.042 *
Heat transfer coefficient (W·m^−2^·K^−1^)	1.07 *
Sorption mass moisture (%)	20 *
Maximum temperature (°C)	170 *

* According to manufacturer’s (Naturwool Ltd., Prague, Czech Republic) data sheets.

**Table 2:** Types and properties of used resins.

Parameter	Matrix		
	EP	PES	PU
Resin type	EPOX G20	GPE 100	GAFORM R30
Resin/hardener mixing ratio (–)	100/23 *	100/1.25 *	100/100 *
Resin viscosity (mPa·s)	450 * (at 23 °C)	200 * (at 23 °C)	285 * (at 20 °C)
Hardener viscosity (mPa·s)	30 * (at 23 °C)	10 ÷ 20 * (at 23 °C)	150 * (at 20 °C)
Density (g·cm^−3^)	1.00 ÷ 1.05 *	1.12 *	1.09 *

* According to manufacturer’s (Dawex Chemical Ltd., Prague, Czech Republic) data sheets.

**Table 3:** Mean values and corresponding standard deviations of the first resonance frequencies *f_R_*_1_ depending on the sheep wool concentration *W_r_* for three different composite materials loaded with an inertial mass *m_i_* = 90 g.

Material Type	*W_r_* (w.%)	*f_R_*_1_ (Hz)
EP	0	466 ± 21
	3	253 ± 11
	5	174 ± 9
PU	0	339 ± 12
	3	217 ± 9
	5	130 ± 6
PES	0	219 ± 10
	3	154 ± 8
	5	112 ± 5

**Table 4:** Mean values and corresponding standard deviations of the first resonance frequencies *f_R_*_1_ depending on the wool thickness *t* for pure sheep wool loaded with an inertial mass *m_i_* = 10 g.

Material Type	*t* (mm)	*f_R_*_1_ (Hz)
Sheep wool	10	115 ± 5
	20	51 ± 3
	30	28 ± 2

**Table 5:** Light transmission coefficient *τ* of the EP/sheep wool and PES/sheep wool composites.

Material Type	*W_r_* (w.%)	*τ* (–)
EP	0	0.69 ± 0.03
	3	0.43 ± 0.02
	5	0.37 ± 0.02
PES	0	0.72 ± 0.04
	3	0.58 ± 0.03
	5	0.53 ± 0.03

**Table 6:** Light reflectance *ρ* of the individual surfaces in the reference room.

Surface	*ρ* (–)
Walls	0.94
Ceiling	0.94
Floor	0.65
Brown door	0.16

**Table 7:** Mathematically simulated values of the daylight factor *DF* in the reference room for the tested polymer samples.

Material Type	*W_r_* (w.%)	*DF_min_* (%)	*DF_m_* (%)	*DF_max_* (%)
EP	0	8.8	4.4	1.5
	3	5.3	2.7	0.9
	5	4.5	2.3	0.8
PES	0	8.5	4.3	1.4
	3	7.1	3.6	1.2
	5	6.5	3.3	1.1

**Table 8:** Physical and mechanical properties of selected NFs [58,63].

Group of NFs	Type of NFs	Density (g·cm^−3^)	Tensile Strength (MPa)	Young’s Modulus (GPa)	Elongation (%)
Afs	Sheep wool	1.5–2.0	120–174	1.0–4.8	25–45
	Spider silk	1.34–1.38	25–50	2.0–6.0	10–40
	Chicken feather	0.80–0.89	187	4.6	8
VFs	Cotton	1.21	287–597	6–10	2–10
	Flax	1.38	343–1035	50–70	7
	Jute	1.23	187–773	20–55	1.5–3.1
	Hemp	1.47	580–1110	30–60	1.6–4.5
	Pineapple	1.50	170–1627	60–82	1.0–3.0
	Sisal	1.20	507–855	9–22	1.9–3.0
	Kenaf	1.20	295–930	22–60	2.7–6.9
	Bamboo	0.6–1.1	270–862	18–89	1.6–8.0
MFs	Basalt	2.7	2130	93	2

## Data Availability

Data are contained within the article.
